# CD4+ T Cells Predict Relapse in Pemphigus Vulgaris Treated With Rituximab: A Retrospective Study

**DOI:** 10.1111/odi.70234

**Published:** 2026-04-08

**Authors:** Simone Liguori, Noemi Coppola, Elvira Ruoppo, Federica Canfora, Gabriele Mignogna, Ilaria Mormile, Antonia Fiore, Giuseppe Castaldo, Michele Davide Mignogna, Stefania Leuci

**Affiliations:** ^1^ Department of Neuroscience, Reproductive and Odontostomatological Sciences University of Naples “Federico II” Naples Italy; ^2^ Department of Translational Medical Sciences University of Naples “Federico II” Naples Italy; ^3^ Clinical and Experimental Cytometry Unit, Centre for Advanced Biotechnology Franco Salvatore, CEINGE Naples Italy

**Keywords:** flow cytometry, pemphigus vulgaris, relapse, Rituximab, T cells

## Abstract

**Objective:**

This study evaluated the CD4^+^ T‐cell role in mediating post‐Rituximab Pemphigus vulgaris (PV) relapse, comparing CD4^+^ count and CD4^+^/CD20^+^ ratio between patients who achieved remission and those who relapsed.

**Methods:**

The clinical course of 27 PV patients treated with Rituximab was evaluated after a 32‐month median follow‐up. CD4^+^ and CD20^+^ counts and CD4^+^/CD20^+^ ratio were longitudinally collected at treatment start date (T0), at 2‐month intervals after Rituximab, until B‐cell repopulation, at B‐cell repopulation, at 6‐, 12‐, and 24‐month intervals after repopulation, and at the end of follow‐up or at relapse.

**Results:**

Patients were administered Rituximab as adjuvant therapy: 16 (59%) relapsed while 11 (41%) achieved clinical remission. Higher CD4^+^ count (*p* = 0.02*) and CD4^+^/CD20^+^ ratio (*p* = 0.004**) were found at B‐cell repopulation in patients experiencing remission. Moreover, a significant difference (*p* = 0.002**) in post‐repopulation CD4^+^ T‐cell course was found between groups, with patients in clinical remission reporting a mean decrease of 233.5 cells/μL during follow‐up and relapsing patients experiencing a mean increase of 539.4 cells/μL and reaching the maximum CD4^+^ value at relapse.

**Conclusions:**

Lower CD4^+^ T‐cell value at repopulation and increasing post‐repopulation CD4^+^ T‐cell count were predictive of disease relapse suggesting a time‐dependent role of CD4^+^ T cells in post‐Rituximab PV reactivation.

## 1 Introduction

### 1.1 General

Pemphigus vulgaris (PV) is a severe and potentially life‐threatening autoimmune blistering disease that predominantly affects women and adults in their 40s and 50s. It is a rare disease, with prevalence rates ranging from 30 to 0.38 per 100,000 people per year, and an incidence between 5 and 0.098 per 100,000 people per year, depending on ethnic background (Rosi‐Schumacher et al. 2023).

While the exact cause of PV remains unclear, both genetic factors and environmental triggers such as UV exposure, stress, physical trauma, diet, and medication use are believed to contribute to the onset or the exacerbation of the disease (Lim et al. 2022). The pathogenesis of PV has been largely elucidated. Blister formation is induced by the loss of intraepithelial cell–cell adhesion that is mediated by IgG autoantibodies (Abs) targeting the desmosomal glycoproteins desmoglein 1 (Dsg1) and desmoglein 3 (Dsg3) (Kasperkiewicz et al. 2017). In a proposed multipathogenic model, IgG Abs disable Dsg‐mediated intra‐epithelial interactions through steric hindrance and alter the salvage pathways of keratinocytes leading to acantholysis and to the loss of epidermal integrity (Ahmed et al. 2016).

### 1.2 The Role of T Cells in PV Pathogenesis

Although PV is considered a prototype of a B‐cell mediated disease, T cells play a crucial role in the initiation and perpetuation of the autoimmune pathogenic mechanism (Lee et al. 2023). In recombination activating gene 2‐knockout mice (Rag2−/−) PV phenotype was found to be induced only when both Dsg3‐specific B cells and T cells were injected (Tsunoda et al. 2002). Moreover, specific human leucocyte antigen (HLA) class II alleles have been associated to increased PV susceptibility. HLA‐DRB polymorphisms DRB1*04, DRB1*08, and DRB1*14 have been frequently reported in PV patients (Yan et al. 2012) and HLA‐DRB1*04:02‐T cells were found to induce pathogenic IgG Abs' production in a humanized HLA‐DRB1*04:02‐transgenic mice (Eming et al. 2014). In human cells studies autoreactive Abs' production was found to be completely abolished by CD4+ T‐cell depletion (Nishifuji et al. 2000). Furthermore, increased number of both autoreactive Th1 and Th2 cells was described in PV patients in different stages of the disease (Veldman et al. 2003) and elevated serum levels of Th2 cytokines IL‐4, IL‐5, IL‐6, and IL‐10 have been found in active disease (Amber et al. 2013). More recently, increased concentration of Th17 and Tfh17 were described in PV patients and, through transcriptome sequencing and quantitative real‐time PCR, IL‐17A signaling was found to be primarily implicated in B‐cell stimulation and Abs production (Holstein et al. 2021). Finally, a lower in vitro suppressive activity of T regs was reported in PV (Su et al. 2020) and Foxp3‐expressing T regs and Dsg3‐specific T regs were found to be decreased in patients compared to controls and healthy carriers of HLA‐DRB1*04:02 (Abd El‐Magid et al. 2022; Veldman et al. 2004).

### 1.3 The Effect of Rituximab (RTX) on B Cells

In patients recalcitrant to conventional immunosuppressive therapy (CIST), B‐cell‐targeted biologic therapies have proven effective as second‐line treatments (Joly et al. 2017; Werth et al. 2021). Rituximab (RTX), a chimeric monoclonal antibody targeting B‐lymphocytes' surface marker CD20, was the first biologic therapy approved for PV. The post‐RTX remission rate ranges from 47% to 89.5%, with mean times to disease control and complete remission being 1.1 and 5.8 months, respectively (Ly et al. 2023; Wang et al. 2015). Nonetheless, RTX‐induced B‐cell depletion has frequently short‐term efficacy, and relapse rates following B‐cell repopulation can range from 18% to 52% (Ly et al. 2023). The current strategy for the prognostic management of PV patients treated with RTX is based on the assay of autoreactive Abs titres and on the evaluation of post‐RTX CD20+ B‐cell course. Late B‐cell repopulation occurring more than 12 months after treatment was found to be associated with lower relapse probability, with only 10% of patients experiencing disease reactivation. Furthermore, increased levels of anti‐Dsg1 and anti‐Dsg3 Abs after B‐cell repopulation were found to be associated with a higher risk of relapse, with proportional hazard ratios of 6.40 and 5.15, respectively (Albers et al. 2017). However, both Abs titres and CD20+ B‐cell assessments have shown limited sensitivity, indicating the need for novel prognostic biomarkers in PV patients' monitoring.

### 1.4 The Effect of B‐Cell Depletion on T Cells

The effect of B‐cell depletion on T‐cell course has not been thoroughly investigated. Post‐RTX T‐cell concentrations were assessed for the first time by Mouquet et al. (2008) and Eming et al. (2008) who reported no significant variation in T‐cell number and percentage after treatment and no association between post‐RTX T‐cell frequency and patients' clinical course. However, a significant decrease in Dsg‐specific CD4+ T‐cell number was observed, with subsequent increase or further decrease in patients who respectively experienced relapse or complete remission. Contrarily, Albers et al. (2017) described a correlation between post‐RTX number and frequency of T cells and patients' response to treatment. Lower T‐cell concentrations, in fact, were associated with a 25‐fold increased relapse risk and, at multivariable analysis, any CD4+ value increase of 200 cells/μL was found to decrease the hazard ratio for relapse by 35%.

### 1.5 Aims

This study aimed to evaluate post‐RTX CD4+ T‐cell course in relation to patients' clinical response investigating T‐lymphocytes' role in PV flare‐up. The accuracy of novel cytofluorimetric biomarkers to use in patients' prognostic management is also assessed and any correlation between post‐RTX T‐cell immunophenotyping and patients' baseline characteristics is ruled out.

## 2 Methods

### 2.1 Patients

This study was conducted according to the World Medical Association Declaration of Helsinki and approval was obtained by the Ethical Committee of the University of Naples Federico II (protocol number 69/19). Written informed consent was signed by each participant. A total of 27 patients were recruited. A diagnosis of PV was confirmed by mucosal histopathology and direct immunofluorescence of tissue‐bound Abs or serum assay of anti‐Dsg3 and anti‐Dsg1 antibodies through Enzyme Linked Immunosorbent Assay (ELISA). All the patients were treated with CIST receiving 1 mg/kg/day of prednisone with or without 100 mg/day of azathioprine for almost 4 weeks and were administered RTX as adjuvant therapy for not responding to CIST (CIST‐recalcitrant) or relapsing during steroids' tapering (CIST‐relapsing). The following were used as exclusion criteria: (i) neoplastic or immune‐mediated comorbidity, (ii) previous immune‐modulating therapies other than CIST, (iii) multiple RTX cycles. All the patients received RTX at the Oral Medicine Unit of the University of Naples Federico II between 2017 and 2021. Among the group of patients who completed the therapeutic cycle by 2021, only those (*n* = 27) for whom baseline data were completely available were included in the study. As the dosing protocol used in RTX treatment changed over time, in this study patients were administered RTX with different regimens: 8 patients were treated with a modified protocol (MP) consisting of 8 weekly and 4 successive monthly infusions of 375 mg/m^2^ of BSA, while for 9 and 10 patients were used the LP (Grillo‐López et al. 1999) and the RAP (Smolen et al. 2007) respectively.

### 2.2 Post‐Rituximab Clinical Evaluation

Post‐RTX clinical course was evaluated after a 32‐month median follow‐up and late endpoints were defined according to the consensus statement (Murrell et al. 2008). Relapse (R) was described as the appearance of new, non‐self‐limiting lesions while clinical remission off therapy (CR) as the absence of new or established lesions when the patient was off all systemic medications. Patients' clinical history was collected including baseline disease phenotype, CIST duration, B‐cell repopulation time (i.e., the time from the last infusion of the treatment regimen to the reappearance and increase of B cells up to 5 cells/μL), relapse time (i.e., the time from B‐cell repopulation to R) and clinical remission time (i.e., the time from the treatment start date (T0) to CR). Clinical disease activity was assessed at the onset, at T0 and at the end of follow‐up or at relapse using the Pemphigus Disease Area Index (PDAI) that is widely used for stratifying PV patients based on the extension of mucosal and cutaneous involvement (Hébert et al. 2019). A more detailed description of PDAI assessment is provided as Supporting Information.

### 2.3 Immunophenotyping Assessment

**FIGURE 1:**
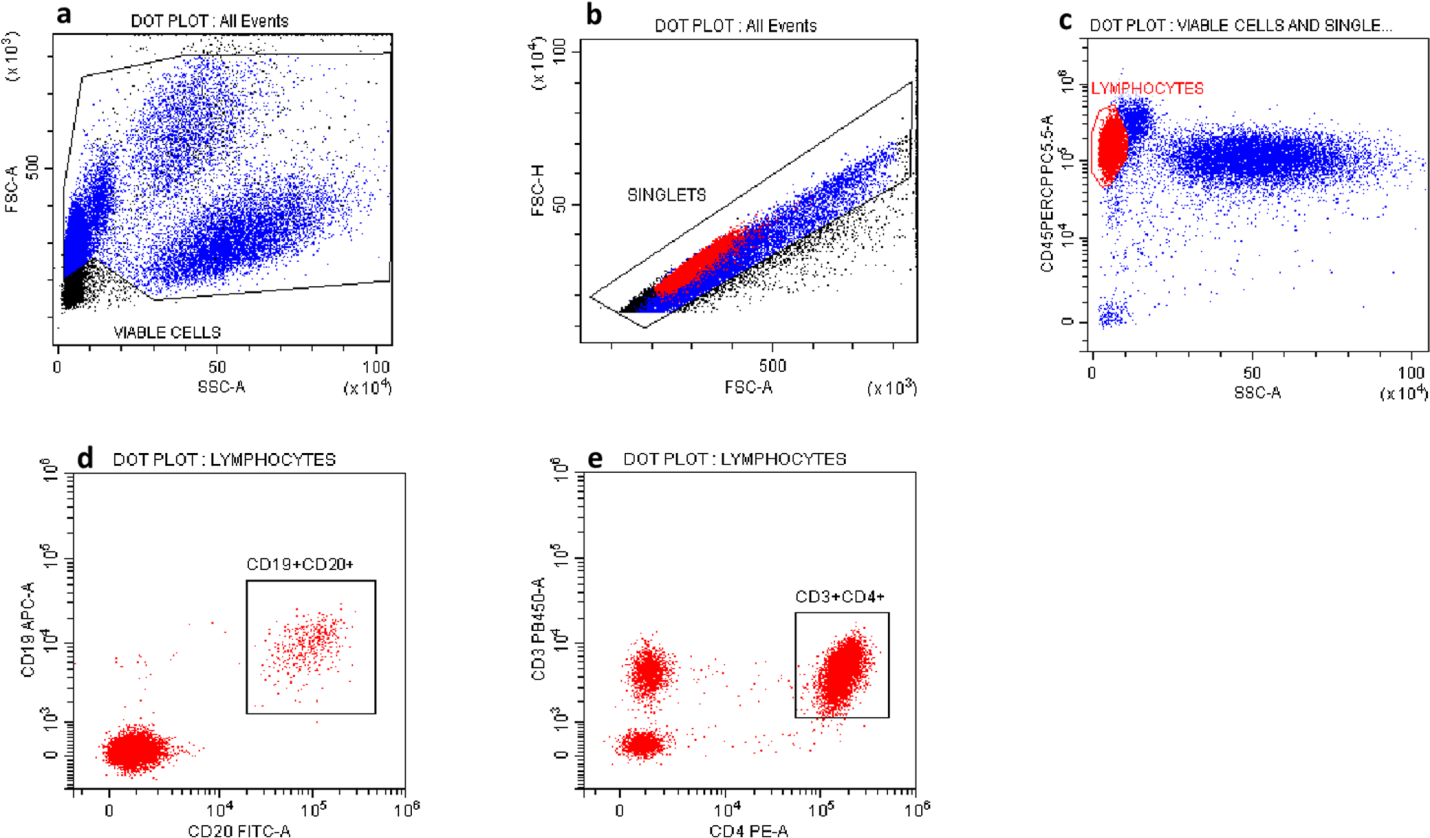
PBMC immunophenotyping. (a–c) Lymphocytes' gating: Live cells were selected based on physical parameters FSC‐A vs. SSC‐A. (d) B‐cell analysis. (e) T‐cell analysis. PBMC, peripheral blood mononuclear cells.

Peripheral blood mononuclear cells (PBMC) were collected from each patient at T0 and after RTX: at 2‐month intervals until B‐cell repopulation, at B‐cell repopulation, at 6‐, 12‐, and 24‐month intervals after repopulation and at the end of follow‐up or at relapse. CD4+ and CD20+ cell numbers were evaluated. CD4+/CD20+ ratio was additionally measured to assess whether the proportion between T and B cells was maintained, despite any potential difference in total lymphocyte subsets values. The cut‐off of 800 cells/μL was used to distinguish high and low levels of T cells (Albers et al. 2017). For CD4+/CD20+ ratio, instead, the threshold value of 20 was established post hoc. The gating strategy was as follows: lymphocyte cells were gated by using CD45 versus SSC‐A identifying 50,000 events (Figure 1). No viability dyes were used as live cells were selected based on physical parameters FSC‐A versus SSC‐A. Doublets and debris were excluded upon analysis and leukocytes gate. Additional technical details of the immunophenotyping procedure are provided as Supporting Information.

### 2.4 Statistical Analysis

Median ± IQR was used for descriptive statistics. The distribution of categorical data was investigated through Fisher's Exact test while Mann Whitney *U* test was used to compare non‐normal numerical data between groups. Univariate survival analysis was conducted through Cox regression and graphically described through the Extended Kaplan Meyer plot. Shapiro Wilk test was used to assess data normality while variance homogeneity was evaluated using the Levene test. A two‐sided analysis was performed and *p*‐value < 0.05 was considered statistically significant. SPSS version 29.0.1.0 (IBM Corporation, Armonk, NY, USA) and GraphPad Prism 10.4.0 (GraphPad Software, Boston, Massachusetts USA) were the software used for statistical analysis and graphing.

## 3 Results

### 3.1 Clinical Characteristics

Table 1 describes the demographic and baseline characteristics of the patients. Median age at PV onset was 53 ± 20 and female to male ratio was 1.45/1. Mucous membranes were the only sites affected in 13 patients while 14 patients had muco‐cutaneous disease. Median Pemphigus Disease Area Index (PDAI) score at baseline was 22 ± 31 and the most affected mucous and cutaneous sites were oral cavity (100%) and conjunctiva (38.5%) and scalp (34.6%), respectively. Anti‐Dsg1 and anti‐Dsg3 titres in median values were 51.1 and 100 RU/mL, respectively. All the patients were treated with CIST, 4 (15%) patients received 1 mg/kg/day of prednisone while 23 (85%) patients were treated with prednisone in combination with azathioprine 100 mg/day. Median CIST duration was 7 ± 9 months, 14 (52%) patients achieved disease control but relapsed during steroids' tapering; 13 (48%) patients, instead, were recalcitrant. No differences in demographics and baseline characteristics, including CIST type and length and therapeutic response to CIST, were found between patients who experienced relapse (R) after RTX and those who were in clinical remission off therapy (CR) at the end of the study. Furthermore, no correlations were found between baseline values of autoantibodies titres and disease activity score nor between clinical characteristics at baseline and post‐RTX T‐cell course.

**TABLE 1:** Demographics and baseline clinical characteristics.

		Total	CR	R	
F/M (N/N)		1.45/1	1.75/1	1.66/1	ns
Age (median ± IQR)		53 ± 20	53 ± 15	49.5 ± 20.25	ns
Disease type, *N* (%)	Mucous	13 (48)	5 (45)	8 (50)	ns
	Muco‐cutaneous	14 (52)	6 (55)	8 (50)	
CIST type, *N* (%)	Prednisone	4 (15)	—	4 (25)	ns
	Prednisone + Azathioprine	23 (85)	11 (100)	12 (75)	
CIST duration^a^ (median ± IQR)		7 ± 9	10.5 ± 9	7 ± 8	ns
PDAI T0 (median ± IQR)		22 ± 31	30 ± 33	16 ± 19	ns
Anti‐Dsg1 RU/mL (median ± IQR)		51.1 ± 61.2	49.6 ± 64.4	52.7 ± 43.4	ns
anti‐Dsg3 RU/mL (median ± IQR)		100 ± 53.3	100 ± 63.9	100 ± 105.5	ns

Abbreviations: CIST, conventional immunosuppressive therapy; CR, clinical remission; IQR, interquartile range; ns, non‐significant; PDAI, Pemphigus Disease Area Index; R, relapse.

^a^ Duration (months).

### 3.2 Rituximab and Post‐Therapy Course

CIST recalcitrant and relapsing patients were treated with RTX using three different therapeutic regimes: 8 patients were administered a modified protocol (MP), while for 10 and 9 patients were followed the rheumatoid arthritis protocol (RAP) and the lymphoma protocol (LP) respectively. Median pre‐RTX PDAI was 13 ± 8. All the patients achieved disease control and median time to CR was 7 ± 7.5 months. B‐cell repopulation occurred with a median time of 7 ± 6 months from the last infusion of the treatment cycle and no patients relapsed during B‐cell depletion. Sixteen (59%) patients relapsed with a median time from B‐cell repopulation of 5 ± 6.5 months. Median PDAI at R was 7 ± 4.5 and most frequently patients experienced a mucous flare‐up with oral (85%) and nasal (21.5%) involvement. Eleven (41%) patients, instead, were in CR at the end of a 32‐month median follow‐up. No differences between R and CR were found in RTX regimen nor in post‐RTX CR time and B‐cell repopulation time and RTX timing and protocol was not found to impact post‐RTX immune recovery. Table 2 shows RTX‐related patients' clinical characteristics.

**TABLE 2:** Clinical characteristics related to RTX treatment.

		Total	CR	R	
RTX regimen, *N* (%)	Modified protocol	8 (30)	1 (9)	7 (44)	ns
	Rheumatoid arthritis protocol	10 (37)	6 (55)	4 (25)	
	Lymphoma protocol	9 (33)	4 (36)	5 (31)	
Repopulation time^a^ (median ± IQR)		7 ± 6	9 ± 9	8 ± 6	ns
Remission time^b^ (median ± IQR)		7 ± 7.5	5 ± 6	6 ± 7.25	ns
Relapse time^c^ (median ± IQR)		—	—	5 ± 6.5	ns
PDAI pre‐RTX (median ± IQR)		13 ± 8	8.5 ± 7.75	12.5 ± 6.5	ns
PDAI relapse (median ± IQR)		—	—	7 ± 4.5	ns

*Note:* —, not applicable.

Abbreviations: CR, clinical remission; IQR, interquartile range; ns, non‐significant; PDAI, Pemphigus Disease Area Index; R, relapse; RTX, Rituximab.

^a^ Time (months) from the last infusion of the treatment regimen to the reappearance and increase of B cells up to 5 cells/μL.

^b^ Time (months) from the treatment start date (T0) to CR.

^c^ Time (months) from B‐cell repopulation to R.

### 3.3 Post‐Rituximab CD4+ T Cells

**FIGURE 2:**
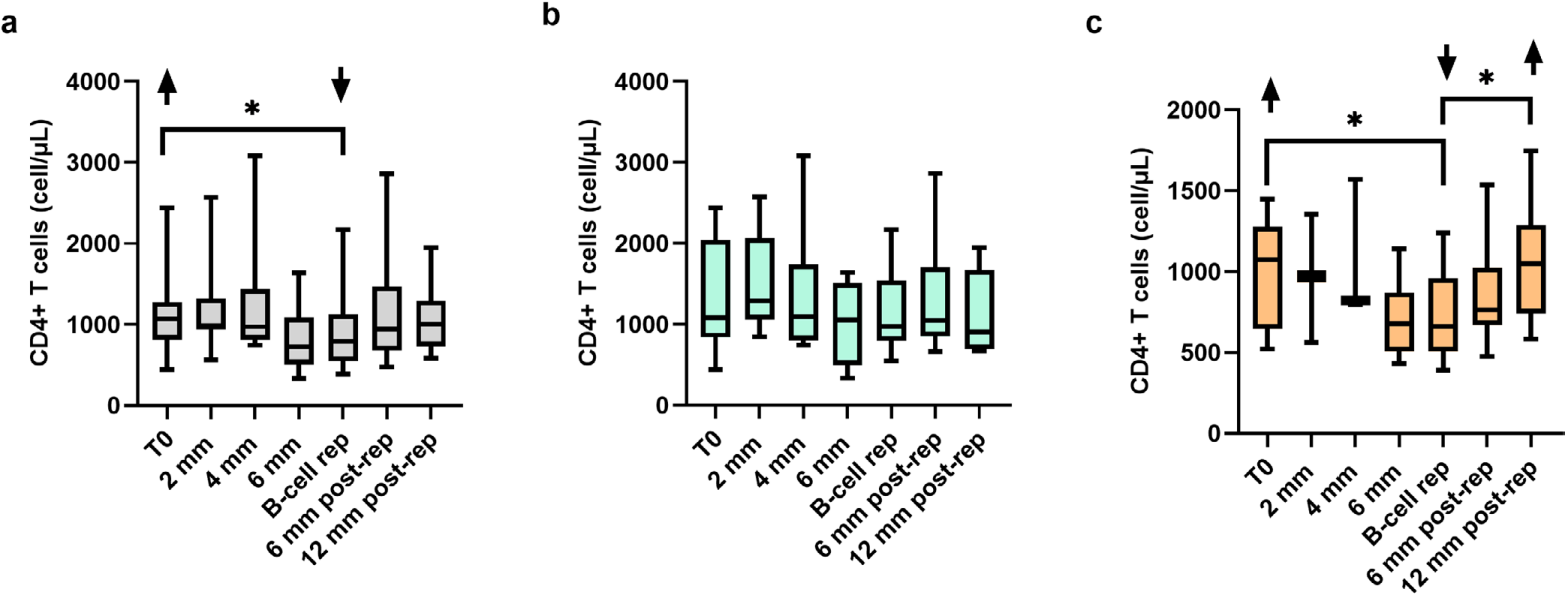
CD4^+^ T cells have a time dependent variation after RTX in relapsing patients. Panel (a) illustrates post‐RTX T‐cell course in the whole sample. A slight decrease in CD4^+^ T‐cell concentration was reported at B‐cell rep compared to baseline. Panel (b) shows post‐RTX CD4^+^ T‐cell course in CR group. CD4^+^ T‐cell counts remained unchanged along the follow‐up. Panel (c) describes post‐RTX T‐cell course in R group. CD4^+^ cells decreased after RTX reaching their minimum value at B cell repopulation and increased following B‐cell reappearance. Results were analyzed by Mann Whitney *U* test. Statistical significance is demonstrated as *p*‐values at the respective time points (*p*‐value < 0.05 was indicated as *). B‐cell rep, B‐cell repopulation; post‐rep, post‐repopulation.

When evaluating the study population as a whole no significant variation was reported in CD4+ T‐cell course between T0 and post‐RTX assessment. Only at B‐cell repopulation a slightly reduction in CD4+ T‐cell count was described and this decrease was statistically significant (*p*‐value < 0.05*) compared to baseline (Figure 2a). Splitting the population and assessing R and CR separately post‐Rituximab CD4+ T‐cell number was found to remain unchanged compared to baseline in CR patients. In R group, instead a stepwise pre‐repopulation CD4+ T cells reduction was reported with T cells reaching their minimal value at repopulation time when CD4+ level was significantly low both compared to baseline and to 12‐month post‐repopulation assessment (*p*‐value < 0.05*) (Figure 2b,c).

### 3.4 B‐Cell Repopulation Immunophenotyping

**FIGURE 3:**
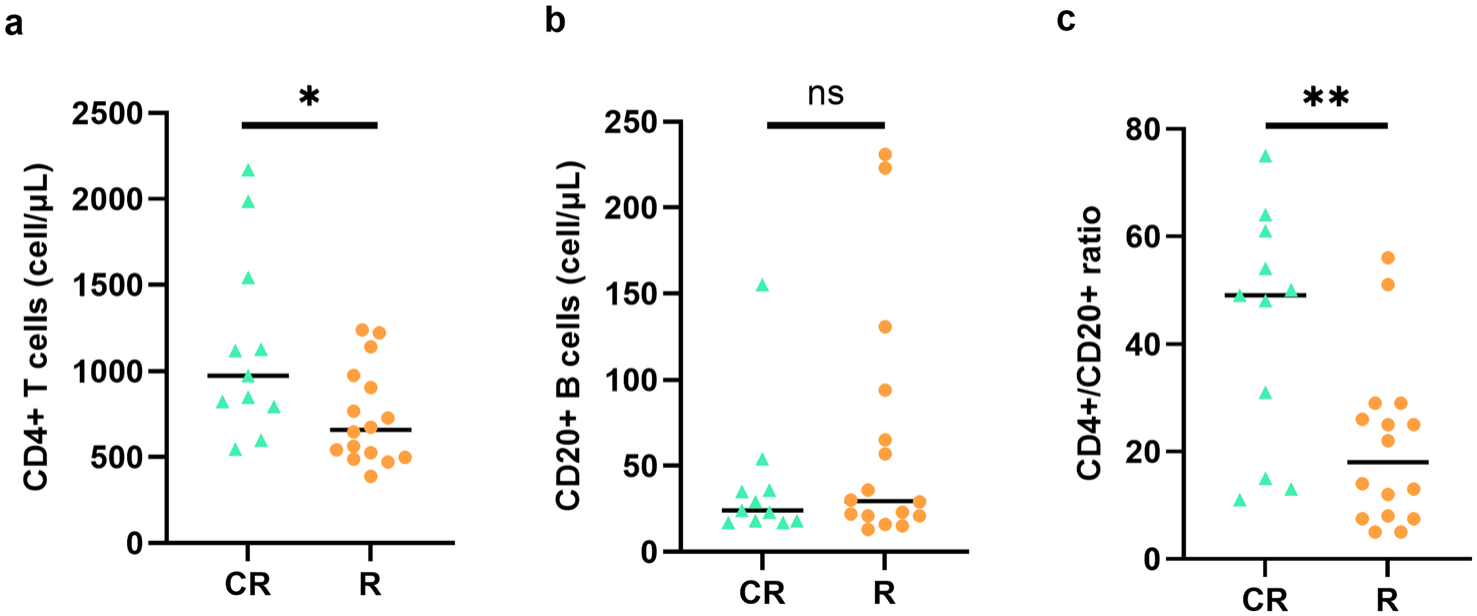
CD4^+^ T‐cell, CD20^+^ B‐cell, and CD4^+^/CD20^+^ ratio distribution at B‐cell repopulation time between CR and R group. (a) CD4^+^ T‐cell count was significantly higher in patients experiencing CR. (b) No difference was found between CR and R in CD20^+^ B‐cell concentration. (c) Significantly higher value of CD4^+^/CD20^+^ ratio was found in CR compared to R. Mann Whitney *U* test was the statistical test used for comparison. Statistical significance is demonstrated as *p*‐values (*p*‐value < 0.05 was indicated as *; *p*‐value < 0.01 was indicated as **). CR, clinical remission; ns, non‐significant; R, relapse.

**FIGURE 4:**
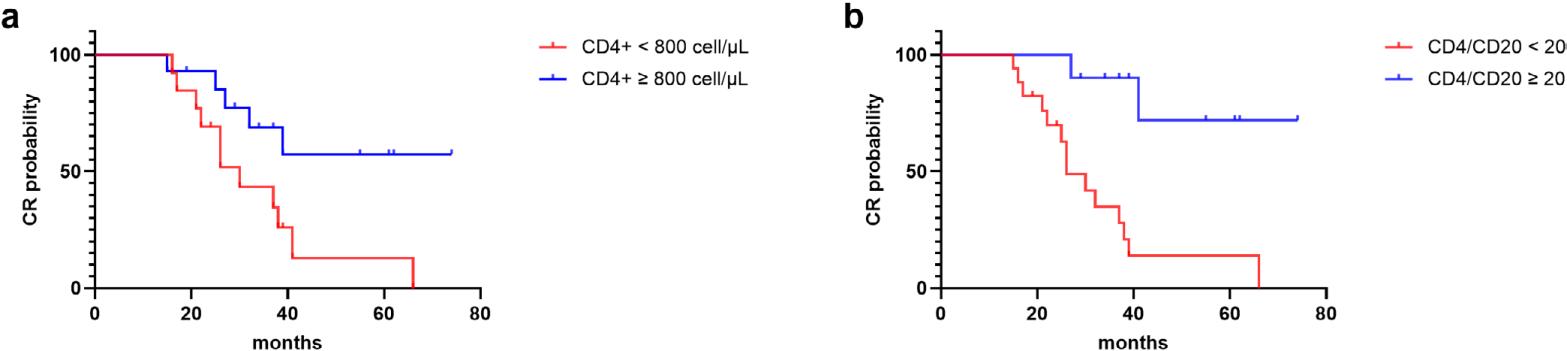
Lower CD4^+^ T‐cell value and CD4^+^/CD20^+^ ratio at B‐cell repopulation time predict relapse. (a) EKM plot illustrates the reported lower relapse risk in patients with CD4^+^ T‐cell count ≥ 800 cells/μL at B‐cell repopulation. (b) CD4^+^/CD20^+^ ratio ≥ 20 was associated with reduced risk of flare‐up as shown in EKM plot. Univariate analysis was conducted using a Cox regression. CR, clinical remission.

At B‐cell repopulation time a statistically significant difference was found in CD4+ T‐cell number (*p*‐value < 0.05*) and CD4+/CD20+ ratio (*p*‐value < 0.01**) between R and CR patients with the latter reporting significantly higher values. CD20+ B cell count was instead comparable between the groups so CD4+/CD20+ ratio discrepancy was exclusively attributable to CD4+ T‐cell value (Figure 3). At univariate analysis CD4+ T‐cell count and CD4+/CD20+ ratio at B‐cell repopulation were found to be highly predictive of patients' clinical response to RTX and CD4+ T‐cell number ≥ 800 cells/μL and CD4+/CD20+ ratio ≥ 20 were associated with a hazard ratio for relapse decreased by 70% (*p*‐value < 0.05*) and 90% respectively (*p*‐value < 0.01**) (Figure 4).

### 3.5 Post‐Repopulation Immunophenotyping

**FIGURE 5:**
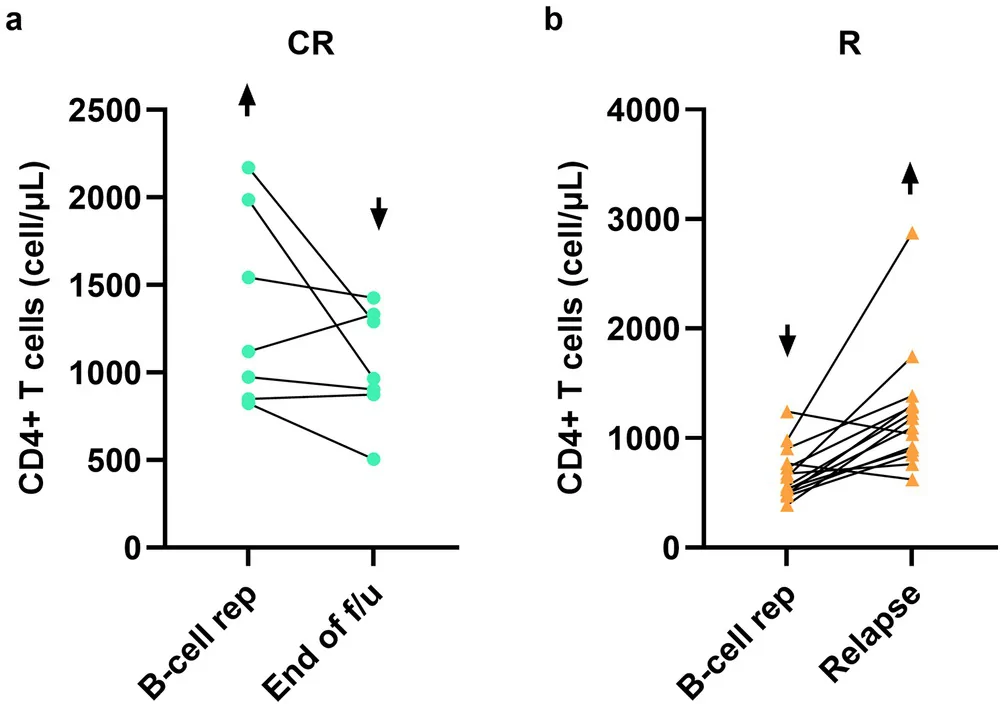
Post‐repopulation CD4^+^ T cells increase in relapsing patients. Panel (a) shows CD4^+^ T‐cell level at B‐cell rep time and at the end of f/u in CR group. CD4^+^ cell count remained unchanged or had a slight reduction, and only two patients experienced a slight increase. Panel (b) illustrates CD4^+^ T‐cell value at B‐cell rep and at disease flare up in R group. All the patients but two, who reported a slight reduction, experienced a significant increase in CD4^+^ T cells. B‐cell rep, B‐cell repopulation; CR, clinical remission; end of f/u, end of follow‐up; R, relapse.

A statistically significant discrepancy between groups was found in post‐repopulation CD4+ T cell course (*p*‐value < 0.01**). CR patients showed, in fact, a mean decrease of 233.5 cells/μL from B‐cell repopulation value to the end of follow‐up while R reported a mean increase of 539.4 cells/μL and reached their highest values at relapse (Figure 5). CD20+ B cells reported, instead, a similar trend in both groups with a mean increase of 121.3 cells/μL in CR and of 144.9 cells/μL in R compared to B‐cell repopulation values.

## 4 Discussion

### 4.1 The Use of RTX in PV Treatment

RTX has proven effective as adjuvant therapy in PV as its combination with CIST has been associated with an increase in remission rate and a reduction in the frequency of steroid‐related side effects (Joly et al. 2007). Furthermore, growing evidence supports the concept that early administration of RTX is linked to a reduced risk of relapse (Balighi et al. 2019; Toosi et al. 2021), and its use as a first‐line therapy has been correlated to a 5‐ and 7‐year disease‐free survival rate of 64% and 62%, respectively (Tedbirt et al. 2024). All the patients recruited in our study were treated with CIST and were administered RTX as second‐line therapy. Curiously, no differences in patients' therapeutic responses were reported according to CIST duration before RTX, and this result was in line with previous papers (De et al. 2020; Kim et al. 2017). Additionally, in accordance with previously reported findings (Zakka et al. 2012), a difference in post‐RTX clinical response was found in relation to RTX regimen, with a higher relapse rate in the group of patients treated with MP and LP compared to the ones treated with RAP. Then our study potentially suggests the RAP protocol as the most effective strategy of RTX administration. However, probably because of the small sample size, no correlation between RTX timing and regimen and patients' clinical response to B‐cell depletion was statistically significant, and this evaluation was beyond the scope of this research that was intended to identify flowcytometric markers of post‐RTX disease flare‐up. Despite the effectiveness of anti‐CD20+ therapy, in fact, even in patients treated with RTX as first‐line therapy, the long‐term relapse rate is still high (Tedbirt et al. 2024), and novel prognostic markers of patients' therapeutic response are needed in post‐RTX clinical follow‐up.

### 4.2 The Effect of RTX on the Immune Response in PV Patients

RTX exerts an effect on both humoral and cellular immune response through different mechanisms. A little percentage of T cells was found to express low levels of CD20 and to be directly affected by B‐cell depletion (Leandro et al. 2006). Moreover, B cells were reported to have antibody‐independent effector functions on T‐cells through antigen presentation and to affect T‐cell activation through cytokine signaling (Martin and Chan 2004). In several autoimmune diseases, a post‐RTX variation in T cells was described. In rheumatoid arthritis, CD4+ cells were found to substantially decrease between 12 and 48 weeks after RTX, and an association was described between T‐cell number and treatment response, with good responders experiencing higher decrease rates compared to non‐responders (Mélet et al. 2013; Piantoni et al. 2015). Similarly, RTX was found to deplete CD4+ IL4+ T cells in skin and peripheral blood of patients with systemic sclerosis, but curiously no effect on CD4+ IFNγ+ and CD4+IL17+ T cells was reported (Antonopoulos et al. 2019). Furthermore, in systemic lupus erythematosus, a decrease in CD4+ cells was described in patients who achieved complete remission after RTX, with a 4‐fold reduction in the expression of costimulatory molecule CD40 ligand (Sfikakis et al. 2005).

This study investigated the effect of B‐cell depletion on CD4+ T cells in PV patients treated with RTX as adjuvant. Differently from recently published papers, this research assessed the general CD4+ population without isolating anti‐Dsg3 specific clones (Hébert et al. 2024; Maho‐Vaillant et al. 2021). This latter technique is, indeed, costly and hard to reproduce and our research was intended to identify easily accessible predictors of post‐RTX clinical course. Furthermore, to the best of our knowledge, this research was the first to assess the correlation between patients' therapeutic response and CD4+/CD20+ ratio by evaluating the accuracy of this novel flowcytometric marker in post‐RTX prognostic evaluation. After the treatment cycle, patients reported a reduction in post‐RTX T‐cell number and at B‐cell repopulation, CD4+ T‐cell count was found to be significantly decreased. This result is in contrast with the studies by Mouquet et al. (2008) and Eming et al. (2008) who reported no effect of B‐cell depletion therapy on T cells in PV. Nonetheless, in these studies, the limited number of relapsing patients could explain the difference from our results. In support of this, we found no variation in CD4+ T‐cell course in patients who experienced clinical remission after therapy while relapsing patients showed a significant decrease in CD4+ cells compared to baseline values. A significant correlation was found in our study between post‐RTX T cell course and patients' clinical response with lower CD4+ cell count and CD4+/CD20+ ratio at B‐cell repopulation strongly associated with increased relapse risk. This was in accordance with the findings by Albers et al. (2017) who reported CD4+ T‐cell values < 800 cells/μL to be suggestive of post‐RTX disease flare up. In line with our results, these authors reported the strongest association between CD4+ cell number and clinical course between 6 and 12 months after RTX cycle that correspond to the time of B‐cell recovery. Higher CD4+ cell values could reflect higher levels of T regs. Increased number and function of CD4+ CD25high cells have been reported in patients with idiopathic thrombocytopenic purpura and myasthenia gravis experiencing clinical remission after B‐cell depletion (Jing et al. 2019; Stasi et al. 2008). Additionally, long‐term RTX efficacy in PV has been associated with the emergence of Dsg3‐specific T follicular regulatory cells highly expressing ICOS, HLA‐DR, and CD25 (Hébert et al. 2024). It is possible, therefore, that peripheral tolerance restoration is required in the early stages of B‐cell repopulation for the recovery of B‐cell physiology. Furthermore, we reported a significant difference in post‐repopulation CD4+ T‐cell course between groups with relapsing patients experiencing an increase in T‐cell count while patients in clinical remission reporting a slight decrease or a steady cell number compared to pre‐repopulation levels. It has been demonstrated that a delayed reappearance of memory B cells and an increased B naïve/B memory ratio after RTX are associated with prolonged clinical remission in patients with PV and other autoimmune conditions (Bergantini et al. 2020; Colliou et al. 2013). Similar findings were described by Iwata et al. (2011) and Sentís et al. (2017) who assessed post‐RTX T‐cell subsets in systemic lupus erythematosus and autoimmune kidney disorders. In these papers, patients in clinical remission reported higher levels of T naïve cells and experienced a delayed amplification of T memory subset while patients who relapsed showed a rapid restoration of memory T cell compartment. Thus, it can be assumed that the rise of CD4+ cells reported after B‐cell repopulation in our cohort of relapsing patients is potentially attributable to an abrupt increase in memory T cells that is likely to be crucial for the perpetuation of the autoreactive immune response and the reoccurrence of the disease.

### 4.3 Limitations

Our study is limited by the retrospective nature and the small sample size that negatively impact sampling and data collection and potentially limit the reproducibility and generalizability of our findings. Nonetheless, patients with both mucosal and mucocutaneous phenotypes, and with both mild and moderate or severe disease activity, were included in this study and our cohort, though limited, could be considered fairly representative of the general population. We investigated post‐RTX CD4+ T‐cells globally without assessing T‐cell subsets. A more complex flowcytometric panel could offer new insights into the immunological mechanisms of post‐Rituximab disease relapse and we have planned a detailed analysis of the post‐RTX course of memory and regulatory CD4+ subsets in order to verify the hypotheses developed in our study. Furthermore, due to the retrospective design of our study, data on post‐RTX autoantibodies course were missing and no correlation between anti‐Dsg1 and anti‐Dsg3 titres, disease activity and CD4+ course after RTX could be investigated. Future prospective studies that more comprehensively assess post‐RTX CD4+ subpopulations and their association with autoantibodies production and patients' clinical course are needed. However, our findings suggest that T cells play a time‐dependent function in PV immunological mechanisms after RTX contributing to the restoration of immune homeostasis or to disease flare‐up. A novel strategy of post‐RTX PV monitoring is also proposed as T‐cell cytofluorimetric assessment could be pivotal in predicting patients' clinical response to B‐cell depletion therapy.

## Author Contributions

**Simone Liguori:** conceptualization, formal analysis, supervision, investigation, methodology, writing – original draft. **Noemi Coppola:** data curation, formal analysis. **Elvira Ruoppo:** investigation, resources. **Federica Canfora:** methodology, data curation. **Gabriele Mignogna:** investigation, formal analysis. **Ilaria Mormile:** methodology, resources. **Antonia Fiore:** methodology, writing – original draft, investigation. **Giuseppe Castaldo:** conceptualization, resources. **Michele Davide Mignogna:** conceptualization, writing – review and editing, supervision. **Stefania Leuci:** resources, supervision, formal analysis, writing – review and editing.

## Funding

The authors have nothing to report.

## Ethics Statement

This study was approved by the Ethical Committee of the University of Naples Federico II (protocol number 69/19) and was conducted in accordance with the World Medical Association Declaration of Helsinki, the local legislation and institutional requirements. The participants provided their written informed consent to participate.

## Conflicts of Interest

The authors declare no conflicts of interest.

## Supporting information


**Data S1:** PDAI calcluation and Immmunophenotyping procedure.

## Data Availability

The original contributions presented in the study are included in the article; further inquiries can be directed to the corresponding author.
