# Supplementary material for: CD4+ T Cells Predict Relapse in Pemphigus Vulgaris Treated With Rituximab: A Retrospective Study
Source: Oral Dis. 2026 Apr 8;32(7):1959–67. doi: 10.1111/odi.70234 (PMC13590902; doi:10.1111/odi.70234)
Supplement: Supplementary file 1 [file ODI-32-1959-s001.docx]

**Supplementary material**

**Pemphigus Disease Area Index calculation**

The PDAI activity score ranges from 0 to 250 points divided into 120 points for skin, 10 for scalp, and 120 for mucosal involvement. Skin activity is assessed across 12 anatomical sites, with each site scored based on the number and size of lesions from a score of 0 that indicates no lesions to a score of 10 that is assigned when there are more than three lesions and/or at least one lesion greater than 16 cm, or when the entire area is affected. Scalp activity is scored based on the presence of blisters, erosions, or erythema. A score of 0 reflects no activity and scores increase based on the number of affected quadrants from a score of 1 for one quadrant to a score of 4 for the involvement of the entire scalp. A score of 10 is given if at least one lesion exceeds 6 cm in size. Finally, mucosal activity is evaluated at 12 different mucosal sites. A score of 0 indicates absence of lesions, a single lesion corresponds to a score of 1, while two to three lesions are scored as 2. A score of 5 is assigned when there are more than three lesions or when two lesions are each larger than 2 cm and the maximum score of 10 is used when the entire mucosal area is affected

**Immunophenotyping procedure**

PBMC immunophenotyping was assessed through the following procedure: an aliquot (50 μL) of anti-coagulated ethylenediaminetetraacetic acid (EDTA) whole fresh blood (within 12 hours) was incubated at 4°C for 30 minutes in the presence of appropriate amounts of monoclonal antibodies. In order to effectively remove erythrocytes, the mixtures were then diluted 1:20 in ammonium chloride lysing solution, incubated at room temperature for 10 minutes and finally washed prior to flow cytometric analysis with the DxFlex cytometer (Beckman Coulter, Marseille Cedex 9, France). Samples were analysed on CytExpert software (Beckman Coulter, Marseille Cedex 9, France). The following antigens were analysed: CD4 PE (from BD San Diego, CA, USA), CD8 APCcy7 (from Beckman Coulter, Marseille Cedex 9, France), CD20 FITC (from BD San Diego, CA, USA), CD19 APC (from Beckman Coulter, Marseille Cedex 9, France), CD45 PerCP (from BD San Diego, CA, USA), CD27 HV500 (from BD San Diego, CA, USA), CD3 Pacific Blu (from Beckman Coulter, Marseille Cedex 9, France). The lower level of detection was 10-4 (as such, zero corresponds to a level below 1/10000 cells). Laboratory procedures were performed in accordance with UK-NEQAS quality standards (https://ukneqas.org.uk/).
